# The acceptability judgment of Chinese pseudo-modifiers with and without a sentential context

**DOI:** 10.1371/journal.pone.0219896

**Published:** 2019-07-18

**Authors:** Tao Gong, Lan Shuai, Yicheng Wu

**Affiliations:** 1 Center for Linguistics and Applied Linguistics, Guangdong University of Foreign Studies, Guangzhou, China; 2 Educational Testing Service, Princeton, New Jersey, United States of America; 3 Department of Linguistics and Translation, Zhejiang University, Hangzhou, China; Nankai University, CHINA

## Abstract

This paper investigates a particular type of non-canonical construction in Mandarin Chinese displaying an apparent semantics-syntax mismatch. We conducted an acceptability judgment experiment on native Mandarin speakers to evaluate whether such sequences could stand out of context as acceptable fragments. Analyses on experimental results revealed that: both semantic and syntactic acceptability of these sequences were significantly lower than those of canonical nominal classifier phrases; whereas if contextualized, the syntactic acceptability of those sequences became similar to that of canonical nominal phrases. This suggests that the non-canonical sequences are grammatically not on the same footing as canonical expressions; and it is the sentential context that makes these sequences appear structurally well-formed. These findings contribute to general discussions on relationship between constituency and grammaticality by demonstrating the gradient nature of grammaticality, and advocate a dynamic perspective in linguistic analysis that looks at a sequence of words in interaction with other elements in a sentence.

## Introduction

A *constituent* in syntactic analysis usually refers to a word or a group of words that could function as a single unit within a hierarchical structure. Constituents as such are largely *phrases* (sequences of words built around a head lexical item and working as a unit in a sentence) in many languages. In contemporary linguistics, especially generative linguistics, constituency tests (e.g., fronting, clefting, replacement, ellipsis, passivization, omission and coordination) play a crucial role in identifying constituent structures and analyzing issues concerning language structure [1]. However, as rough-and-ready tools to reveal clues of syntactic structures, the basic rationale for why such tests should and could tap constituent structure remains unclear [2], and different tests are often applied at stages having disparate representations [3]. Therefore, it is no surprise that many constituency tests tend to deliver contradictory results (for detailed discussions, see [4–8] *inter alia*). Such tests could be more appropriately considered structural heuristics rather than structural diagnostics [9].

This paper aims to discuss the relationship between constituency and grammaticality by examining a particular type of non-canonical classifier constructions in Mandarin Chinese. The reason to choose Mandarin Chinese is that this representative classifier language serves as a good test bed to evaluate grammatical properties and functions of numeral classifiers [10,11]. In Mandarin Chinese, lexical categories can in general be modified by numeral classifiers: Specifically, a nominal can be modified by a nominal classifier as in example (1) in Table 1, a verb by a verbal or temporal classifier as in (2) and an adjective by a measural classifier as in (3) (note that ‘CL’ stands for ‘classifier’ throughout the paper).

**Table 1:** Examples of classifiers in Mandarin Chinese.

(1) a. *yi shou ge*	(2) a. *xie liang bian*	(3) a. *san mi chang*
one CL song	write two CL: time	three CL: meter long
‘a song’	‘write two times’	‘three meters long’
b. *wu pi ma*	b. *zhu san nian*	b. *shi yingchi gao*
five CL horse	live three CL: year	ten CL: foot high
‘five horses’	‘live (somewhere) three years’	‘ten feet high’

Nominal classifiers, which have the greatest number in Mandarin Chinese according to the *Lexicon of Common Words in Contemporary Chinese* (China State Language Commission, 2008), just describe or pick out the inherent, semantic properties of the entities denoted by nouns and are typically treated as attributives when modifying nouns. In general, nominal classifiers can be classified into two types, viz., sortal classifiers (e.g., *shou* in (1a) and *pi* in (1b)) which are usually used with count nouns, and mensural classifiers (e.g., *bei* ‘cup’ in *yi bei jiu* ‘one CL: cup wine’ and *wan* ‘bowl’ in *liang wan tang* ‘two CL:bowl soup’) which are used with mass nouns. As for temporal and verbal classifiers, they are generally treated as adverbials when modifying verbs. The main difference between them is that, by their names, a temporal classifier is used to describe the duration of length of the action or event denoted by a verb, whereas a verbal classifier is used to count the times of the action or event denoted by a verb.

Yet interestingly, it has long been observed in the literature [12,13] that apart from the canonical nominal classifier constructions like those in (1a-b) in Table 1, temporal and verbal classifiers appear also able to modify nominal expressions, as in the two examples in Table 2 (note that ‘ASP’ stands for ‘aspect’ throughout the paper):

**Table 2:** Examples of temporal (1) and verbal (2) classifiers.

(1) *Taotao qi le wu tian ma*.	(2) *Lanlan chang le liang ci ge*.
Taotao ride ASP five CL: day horse	Lanlan sing ASP two CL: time song
‘Taotao did horse-riding for five days.’	‘Lanlan sang a song two times.’

Syntactically speaking, the temporal classifier *tian* ‘day’ in example (1) in Table 2 and the verbal classifier *ci* ‘time’ in example (2) in Table 2 appear to modify the bare nouns *ma* ‘horse’ and *ge* ‘song’, respectively. Yet semantically speaking, they actually quantify the events expressed by the respective verb phrases *qi ma* ‘ride horse’ in example (1) in Table 2 and *chang ge* ‘sing song’ in example (2) in Table 2. Given the semantic mismatch between the temporal/verbal classifier and the nominal in sentences like examples (1) and (2) in Table 2, we treat these combinations as non-canonical expressions vis-a-vis the [nominal classifier + bare nominal] combinations which are canonical expressions. Here, a question that naturally arises is whether such non-canonical sequences (*wu tian ma* ‘lit. five CL: day horse’ and *liang ci ge* ‘lit. two CL: time song’) should be treated as *phrasal constituents*. Whether in traditional grammarians’ works (e.g., [14–17]) or contemporary linguistic studies (e.g., [18]), these sequences have been consistently and explicitly considered to be “pseudo-attributives” on the grounds that the pre-nominal temporal classifier is actually not used to modify the bare nominal but the whole VP formed by the verb and the bare nominal as in example (1) in Table 2, and the verbal classifier is used to modify the verb as in example (2) in Table 2. Also, it is generally agreed among these linguists that the non-canonical constructions at issue are highly productive, for the simple reason that they display a salient property, namely, schematicity formed by a temporal or verbal classifier + a bare nominal. Interestingly, some generative linguists (e.g., [19]) argue against the treatment of the non-canonical sequences as pseudo-attributive constructions, claiming that such sequences, though combined in a non-canonical fashion, should be treated as *noun phrases* when appearing in *a pre-verbal position*, or *verb phrases* when appearing in *a post-verbal position*.

Perhaps the most straightforward way to test whether a sequence of words is a phrasal constituent is to evaluate directly whether the sequence can stand *out of context* as a proper phrase [5]. Regarding the temporal or verbal classifier phrases as in Table 2, the test hinges on whether native Mandarin speakers deem such sequences acceptable when presented without context; if so, such sequences should be justifiably regarded as constituents, since they match our intuitive notion of what words can go together to form phrases. Experimental studies employing such tests can evaluate linguists’ intuitions or theories about complex semantic or syntactic issues. As shown in many recent studies [20–25], experimental studies as such have contributed to theoretical linguistics by validating whether individuals have access to abstract linguistic knowledge as claimed by theoretical linguists, or whether the responses of individuals to specially-designed sentences follow the predictions made by linguistic theories.

In linguistic studies, acceptability judgment of a sentence is based upon whether it is produced and/or interpreted in accordance with the grammatical rules of a language. In principle, grammaticality is purely determined by relevant syntactic knowledge. However, except for nonsensical (meaningless, nor existent or statistically probable in real language corpora) expressions [26], the syntactic components of linguistic expressions would unavoidably involve semantic facts or constraints, and at least some semantic information could impact the grammaticality of linguistic expressions whose syntactic structures and semantic contents may not be compatible [27–32]. In this sense, in order to comprehensively evaluate whether a sequence of words forms a syntactically proper constituent, one needs to take into account both syntactic and semantic acceptability of that sequence. Syntactic acceptability here is based mainly on the surface structure of a sequence, whereas semantic acceptability is determined primarily by the truth value of the proposition expressed by that sequence in relation to the nature of the world, the appropriateness of using it in various contexts, and so on. As has been discussed in recent literature (see [33] inter alia), comparison between the two types of acceptability helps locate the major determinant of (un)grammaticality of a sequence: for instance, if the semantic acceptability of two sequences is similar, their syntactic acceptability may contribute to the judgment of their (un)grammaticality; similarly, if the syntactic acceptability appears similar, their semantic acceptability can inform (un)grammaticality. In addition, it has been repeatedly shown that semantic information can be referred to in the absence of clear syntactic knowledge and/or processing power in both production and interpretation of infrequent expressions, such as garden path sentences (e.g., [34]) or sentences involving complex clausal structure (e.g., [35]). In these situations, semantic acceptability could function independently of syntactic judgments in support of production and comprehension [36]. This phenomenon has been recorded in both first and second language processing/learning of adults and children (e.g., [36–38]). Finally, unlike categorical grammaticality, acceptability of a sequence is usually a gradient scale [39], yet in practice, the judgment of acceptability usually involves a variety of scales (e.g., a 5- or 7-degree Likert scale).

Taking these into account, we conducted an acceptability judgment experiment to address the grammatical status of the non-canonical [temporal/verbal classifier + N] sequences at issue. During the experiment, we collected, via questionnaires, the syntactic and semantic acceptability of these sequences from native Mandarin speakers. The syntactic acceptability judgment was based primarily on native speakers’ syntactic knowledge on the surface structure of the stimuli, whereas the semantic acceptability judgment was determined mainly by the appropriateness of using the stimuli for interpretation, allowing temporal ignorance of their syntactic structures. To address the influence of linguistic context, we collected syntactic acceptability in two cases, within and without a sentential context. Considering that any sentential context would always induce additional syntactic considerations (e.g., selective simplification of certain elements due to context), the stimuli of semantic acceptability judgment were presented without a sentential context. We used the [nominal classifier + N] sequences as the baseline for fully grammatical (/acceptable) expressions, and the sequences excluding classifiers as the controls for fully ungrammatical (/unacceptable) expressions. The web addresses of the questionnaires can be found in S1 Table.

Our experiment showed that:

1. Without a proper context, the semantic and syntactic acceptability of the non-canonical sequences at issue were much lower than those of the nominal classifier phrases;
2. The semantic acceptability of those sequences appeared to be similar to that of the fully ungrammatical controls;
3. Within a proper context, the syntactic acceptability of those sequences became similar to that of the nominal classifier phrases.

These results serve as telling evidence that the [temporal/verbal classifier + N] sequences are not genuine phrases as the nominal classifier phrases are. More specifically, it is the sentential context that induces the relatively high acceptability of these sequences.

In the following sections, we first describe the experiment, and then report and discuss the results. After that, we summarize the contributions of our study to linguistic studies.

## Materials and methods

The protocol of this experiment was approved by the College Research Ethics Committee of the University with which one of the authors is affiliated to. The methods were carried out in accordance with the approved guidelines from the College Research Ethics Committee. Informed consents were obtained from all participants.

The experiment consisted of two parts:

1. To examine the syntactic acceptability of linguistic sequences involving different types of classifier phrases (henceforth CLPs) with and without a sentential context;
2. To test the semantic acceptability of those CLPs without a sentential context.

The acceptability scores were collected via predesigned questionnaires. Questionnaire filling was conducted in a laboratory setting, where participants were asked to fill in the questionnaire in a quiet room without any interference, and they were monitored throughout the experiment. Following a between-subject design, each participant was asked to fill in only one questionnaire. Unlike other means of questionnaire filling (e.g., online or take-home filling), the laboratory setting as in our experiment helped to greatly eliminate the influence of other uncontrollable factors.

Given that the terms of semantic and syntactic acceptability may be confusing to naïve participants, before questionnaire filling participants were given a brief tutorial on how to judge the acceptability of a linguistic sequence based on its semantic or syntactic aspects. As for the syntactic acceptability judgment, participants were instructed to focus on whether the shown expressions were grammatically acceptable according to their native knowledge of Mandarin, or whether they would like to use such expressions in their everyday conversation. As regards the semantic acceptability judgment, participants were asked to take recourse to the semantics of the shown expressions in support of their interpretation, even though the expressions might seem incongruent with standard grammar of Mandarin or participants were reluctant to make use of such expressions in their everyday conversation. During the tutorial, participants were required to provide explanations to their judgments on a number of example expressions in order to reflect whether they actually referred to syntactic or semantic criteria in their judgments. To avoid familiarity effect, the tutorial did not use any materials in the questionnaires. Semantic acceptability judgments as such might appear unusual compared to those in previous research. However, considering the high degree of flexibility of Mandarin syntax [12], such judgments are actually necessary and informative to distinguish whether a non-canonical expression is syntactically acceptable or it is acceptable simply because its form or its position in a sentence does not greatly affect comprehension.

On average, each participant finished the judgment around 8 minutes. All participants finished their judgments within 15 minutes.

### Materials

We focused on three types of CLPs (note that the classifier in a sequence is underscored, and its gloss is italicized):

1. *Nominal CLPs*, e.g., *san* *ben* *shu* (‘Lit. three *copy* book’) ‘three (copies of) books’;
2. *Verbal CLPs*, e.g., *liang* *yan* *da’an* (‘Lit. two *eye* answer’) ‘(looking at) an answer twice’;
3. *Temporal CLPs*, e.g., *yi* *zhen* *shu* (‘Lit one *moment* book’) ‘(reading) a book for a while’.

In Mandarin Chinese, a nominal CLP doubtless serves as an independent and acceptable phrase by itself since, as has been mentioned in the introduction, a nominal classifier is justifiably used to modify a noun in the sense that it usually describes one of the inherent, semantic properties of the entity denoted by the noun. In the experiment, we designed five questionnaires (four for syntactic acceptability and one for semantic acceptability) to collect syntactic and semantic acceptability of nominal, verbal and temporal CLPs, and evaluated whether verbal or temporal CLPs could also form an acceptable phrase by themselves like nominal CLPs.

For syntactic acceptability, we set up two cases, namely, within context (using CLPs to answer questions) and without (using CLPs in an isolated manner).

Dialogue in the form of question and answer is probably one of the best paradigms of language use, during which the interaction between speaker and hearer continuously creates the proper context for the flow of speech production and interpretation [40–43]. In the case with context, we designed 30 questions, each including one item of the nominal, verbal, or temporal CLPs; in total, these questions contained 10 items of each type. For each question, a set of four answer sequences were provided, three of which involved a particular type of CLP, and the other had no classifiers. The order of the four types of answer sequences in each set was randomized. The participants were asked to judge the syntactic acceptability of each answer sequence based on their knowledge of Mandarin Chinese. There were in total 120 answer sequences (4 × 30). The whole study followed a mixed design (10 items × 3 CLP types × 4 sequence types). The 30 questions and their 120 answer sequences were divided into three questionnaires ((a) to (c)), each containing 10 questions and their 40 answer sequences.

Table 3 shows an example question and its four answer sequences:

Sequence (a) consists of a verb (*qi* ‘ride’) plus a noun-less CLP (this sequence is denoted as *ans_v+CLP-n*). In Mandarin Chinese, such a sequence is an acceptable and simplified usage.
Sequence (b) is either a nominal, verbal, or temporal CLP (in Table 3, it is a temporal CLP, and the sequence is denoted as *ans_CLP*).
Sequence (c) is derived from answer (b) by excluding the noun inside the CLP (the sequence is denoted as *ans_CLP-n*). In Mandarin Chinese, such a sequence is treated as a simplified CLP used to answer questions.
Sequence (d) is also derived from answer (b) by excluding the classifier. Such a sequence is definitely ungrammatical in Mandarin Chinese. It serves as a control (the sequence is marked as *ans_control*).

**Table 3:** An example question and its answer sequences (a)–(d) in the case with context.

Question:	*Zhangsan qi le ji tian ma*?	
	*Zhangsan ride-ASP how many day horse*?	
	‘How many days has Zhangsan ridden a horse?’	
Answers:	(a) *qi le wu tian*	(b) *wu tian ma*
	ride-PFV five day	five CL: day horse
	‘ride (horse) five days’	‘five days horse’
	(c) *wu tian*	(d) *wu ma*
	five day	five horse
	‘five days’	‘five (no CL) horse’

In the case without context, we extracted the 30 *ans_CLP* sequences and the 10 *ans_control* sequences from the case with context. These sequences were randomly grouped into 10 sets. Each set contained a nominal CLP, a verbal CLP, a temporal CLP, and a control, which were presented in an isolated manner without context. The order of these sequences in each set was randomized, and the participants were asked to judge the syntactic acceptability of these sequences (questionnaire (d)).

Table 4 lists the four sequences in an example set. Sequence (a) is a nominal CLP. Sequence (b) a verbal CLP. Sequence (c) is a temporal CLP. To be distinguished from those in the case with context, these sequences are marked as *iso_CLP*. Sequence (d) is the control, denoted as *iso_control*.

**Table 4:** An example of the four sequences in the case without context.

(a) *san pi ma*	(b) *liang yan da’an*	(c) *si tian shu*	(d) *wu shu*
three CL horse	two CL: eye answer	four CL: day book	five book
‘three horse’	‘two-view answer’	‘four-day book’	‘five (no CL) book’

For semantic acceptability (questionnaire (e)), the same setting was employed as in the case without context with respect to syntactic acceptability.

As far as we know, there is no existing database that could provide quantitative information on occurrence rates of various Mandarin expressions. Therefore, we only controlled the occurrence frequencies of the target nouns in those CLPs, ensuring those nouns are frequent. To be specific, all the nouns in those CLPs have relatively high frequencies according to the *Lexicon of Common Words in Contemporary Chinese* (China State Language Commission, 2008): they are all among the top 8,000 high frequency words (according to www.cncorpus.org, the using frequency of these words are no lower than 0.0008%). Note that an expression involving a popular noun does not necessarily indicates that the whole expression is also popular or largely acceptable to native speakers.

It is worth mentioning that the functional morpheme of Mandarin *de* can alternatively occur in the CLPs at issue, e.g., *Taotao qi le wu tian* (*de*) *ma* ‘Taotao ride ASP five CL: day DE horse’. We excluded it in these CLPs to avoid additional complexity caused by this problematic morpheme which is generally considered to have multiple functions depending on different structures where it occurs. A brief discussion of its functions is given in S1 Text.

Considering that acceptability is a gradient scale (see [39] inter alia), participants’ judgments were expressed as an integer score ranging from 1 to 7 (a 7-degree Likert scale). Regarding syntactic acceptability, ‘7’ means that an answer or a standalone sequence is syntactically the most acceptable to participants, whereas ‘1’ means the least so. Regarding semantic acceptability, ‘7’ means that, despite its form, participants can most easily grasp the meaning of a standalone sequence, whereas ‘1’ means the least so.

There are two reasons to extend the traditional 5-degree Likert scale to the 7-degree scale used in this experiment. First, the 7-degree scale is more delicate and better in reflecting the similarity or difference among the acceptable or unacceptable sequences. Second, the 7-degree scale is rich enough to identify inappropriate answers. For syntactic acceptability, the judgments of a participant were deemed inappropriate if s/he scored all the sequences equal or less than five scales, i.e., all the scores were within 1 to 5, 2 to 6, or 3 to 7. This indicated that s/he might either reject all the sequences, including the most grammatical nominal CLPs (scoring them within 1 to 5), accept all of them, including those definitely ungrammatical controls (scoring them within 3 to 7), or treat them as having no essential difference (scoring them within 2 to 6). All this reflected that the participant was either not focused during the experiment or too picky for these sequences. For semantic acceptability, a participant’s judgments were deemed inappropriate if s/he either rejected all of the sequences or accepted all of them by giving either equally lowest or equally highest acceptability scores.

Participants could give identical or similar scores for every sequence in a set. In other words, they do not need to select among the four sequences the most acceptable one. One may, of course, design alternative questionnaires in which only one answer is presented after each question. However, this design would require repeating the same or similar questions four times, thus greatly increasing the lengths of questionnaires, which might annoy participants or induce the lengthy effect (i.e., participants may not be patient or stay focused throughout the experiment, by simply giving identical judgments to sequences appearing at the later part of the questionnaire).

### Participants

One hundred and fifty native Mandarin speakers (78 females, mean age = 25.11, SD = 2.28) volunteered for the syntactic acceptability test. To maintain the same number of participants for each of the four questionnaires, once four participants were available, they were randomly assigned to the four questionnaires, respectively. If the judgments of a participant were deemed inappropriate, those judgments were excluded, and a new participant was assigned to fill the same questionnaire. Thirty-one native Mandarin speakers (16 females, mean age = 23.71, SD = 1.22) volunteered for the semantic acceptability test. If the judgments of a participant were deemed inappropriate, those judgments were excluded. No participants majored in linguistics, psychology or related disciplines. This screening was to prevent participants’ linguistic or psychological knowledge from influencing their performances in the judgments.

Following the abovementioned criterion for (in)appropriate judgments, we excluded the judgments of two participants in the syntactic acceptability test, and those of the remaining 148 participants were used for analysis (37 in each questionnaire). Similarly, the judgments of four participants in the semantic acceptability test were excluded, and those of the remaining 27 participants were used for analysis.

### Data analysis

Table 5 lists all the conditions in this experiment.

**Table 5:** Experimental conditions. Conditions {10} and {14} are controls (marked in grey). Conditions {1}–{10} are in the case with context, and {11}–{14} are in the case without context. Conditions {1}–{14} are in the syntactic acceptability questionnaires, and {11}–{14} are also in the semantic acceptability questionnaire (since in the two types of questionnaires they share the same forms and have no context).

sequence \ CLP	nominal	verbal	temporal
***ans_v+CLP-n***	{1}	{2}	{3}
***ans_CLP***	{4}	{5}	{6}
***ans_CLP-n***	{7}	{8}	{9}
***ans_control***	{10}		
***iso_CLP***	{11}	{12}	{13}
***iso_control***	{14}		

Four statistical analyses were conducted based on the syntactic acceptability scores. First, we performed a two-way repeated measures ANOVA to examine the effects of CLP and sequence types. There were three CLP types (nominal, verbal, and temporal) and four sequence types (*ans_v+CLP-n*, *ans_CLP*, and *ans_CLP-n* in the case with context, and *iso_CLP* in the case without; the form of control was excluded in the ANOVA test since it is definitely unacceptable in terms of syntax, but see discussions of the results). Each phrase type has three levels based on the CLP types. By grouping the answer and isolated phrases, the ANOVA test could evaluate the main effect of the CLP types in general use.

Second, we conducted pairwise T-tests to compare the acceptability scores of the controls and those of other experimental conditions. These tests aimed to detect whether the scores in any other experimental conditions were statistically similar to those of the controls. In the case with context, the scores of the controls (condition {10} in Table 5) were compared respectively with those of conditions {1}–{9} (3 sequence types (*ans_v+CLP-n*, *ans_CLP*, *ans_CLP-n*) × 3 CLP types). In the case without context, the scores of the controls (condition {14}) were compared respectively with those of conditions {11}–{13}.

Third, we conducted post-hoc pairwise T-tests to compare the scores of different CLP or sequence types in each case. As to the CLP types, in the case with context, we compared the scores of each pair of the CLP types in each sequence type. In *ans_v+CLP-n*, comparisons were between conditions {1} and {2}, {2} and {3}, and {1} and {3}, respectively; in *ans_CLP*, comparisons were between conditions {4} and {5}, {5} and {6}, and {4} and {6}; and in *ans_CLP-n*, comparisons were between conditions {7} and {8}, {8} and {9}, and {7} and {9}. In the case without context, since there was only one sequence type (*iso_CLP*), comparisons were between conditions {11} and {12}, {12} and {13}, and {11} and {13}, respectively. As to the sequence types, in the case with context, we compared the scores of the three sequence types in each CLP type. In nominal CLPs, comparisons were between conditions {1} and {4}, {4} and {7}, and {1} and {7}, respectively; in verbal CLPs, comparisons were between conditions {2} and {5}, {5} and {8}, and {2} and {8}; and in temporal CLPs, comparisons were between conditions {3} and {6}, {6} and {9}, and {3} and {9}.

Fourth, we conducted post-hoc pairwise T-tests to compare the scores of the CLP types between the two cases. For nominal CLPs, comparison was between {4} and {11}; for verbal CLPs, comparison was between {5} and {12}; and for temporal CLPs, comparison was between {6} and {13}. These comparisons aimed to reveal the contextual effect on the acceptability of the same CLPs.

Three analyses were conducted for semantic acceptability. First, we performed a one-way repeated measures ANOVA to examine the effects of the CLP type in semantic acceptability. There were four CLP types: nominal, verbal, temporal, and control. The reason of involving the form of control as a CLP type is that, such form, though definitely unacceptable in terms of syntax, might not be so in terms of semantics (see discussions of the results).

Second, we conducted pairwise T-tests to compare the scores of the controls and those of the other three conditions; specifically, comparisons were between conditions {11} and {14}, {12} and {14}, and {13} and {14}, respectively.

Third, we conducted post-hoc pairwise T-tests to compare the scores of different CLP types in each case. Comparisons were between {11} and {12}, {12} and {13}, and {11} and {13}, respectively. Note that the comparisons involving the control type were already conducted in the second step.

In the analyses mentioned above, we performed both by-item and by-subject analyses to balance the effects of CLP items and subjects (participants). In the by-item analysis, for each CLP type, we averaged the scores of all the participants for every sequence involving an item of that CLP type in the questionnaires, thus deriving 10 scores for the 10 items of that type. In the by-subject analysis, for each CLP type, we averaged the scores of the 10 items of that type given by the participants in the questionnaires, thus obtaining 37 (in the syntactic acceptability test) or 27 (in the semantic acceptability test) scores for each participant.

All these analyses were conducted using SPSS v.21.0 (IBM Corp., Armonk, NY, USA) and R (https://www.r-project.org/).

## Results

### Syntactic acceptability test

After the Greenhouse-Geisser correction [40], the two-way repeated measures ANOVA revealed significant main effects of the CLP type (by-item: *F*(2, 18) = 58.847; *p* < .0005; *η*_*p*_^*2*^ = .867; by-subject: *F*(2, 72) = 199.909; *p* < .0005; *η*_*p*_^*2*^ = .847) and the sequence type (by-item: *F*(3,27) = 137.293; *p* < .0005; *η*_*p*_^*2*^ = .938; by-subject: *F*(2.103, 72.472) = 131.717; *p* < .0005; *η*_*p*_^*2*^ = .785), and significant interactions between the two (by-item: *F*(2.846, 25.621) = 48.243; *p* < .0005; *η*_*p*_^*2*^ = .843; by-subject: *F*(2.688, 96.782) = 92.772; *p* < .0005; *η*_*p*_^*2*^ = .720).

Following the Bonferroni correction for multiple comparisons [40], we set the critical *p* value in the T-tests to .001. The pairwise T-tests showed that all the other experimental conditions were significantly distinct from the control condition in both cases (see S2 Table). In addition, the post-hoc pairwise T-tests confirmed that the scores of the three CLP types were significantly different from each other in most comparisons, except those between the nominal and verbal CLPs in *ans_CLP-n*, those between the verbal and temporal CLPs in *ans_CLP-n*, and those between the verbal and temporal CLPs in *ans_v+CLP-n* (see S3 Table). Furthermore, the post-hoc pairwise T-tests revealed that different sequence types were significantly different from each other, except those between nominal *ans_v+CLP-n* and nominal *ans_CLP*, and the post-hoc pairwise T-tests between the cases with and without context also showed significant differences (see S4 Table).

**Fig 1:**
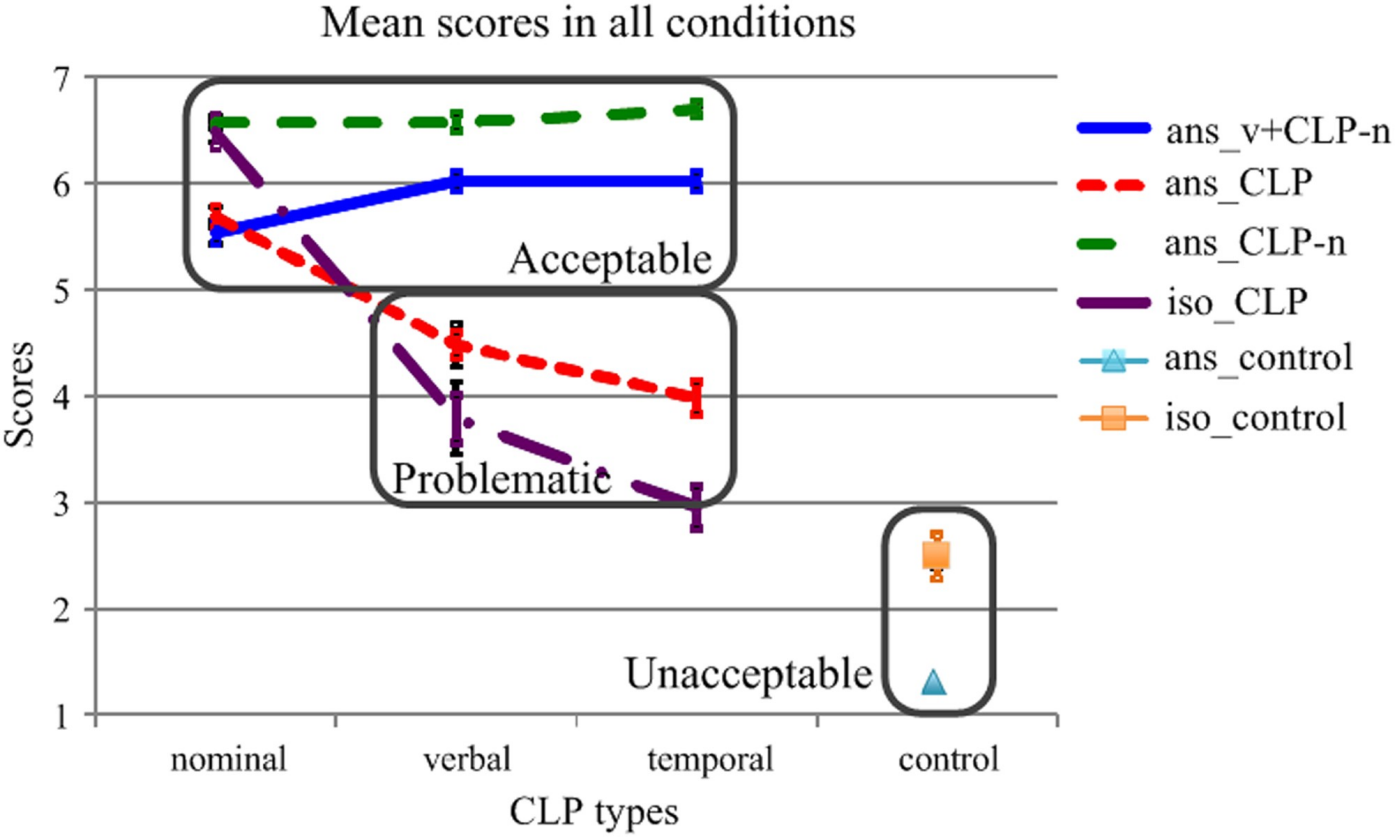
Syntactic acceptability scores in all conditions. The two error bars at each data point mark the SEs of the by-item and by-subject analyses. The three rectangles mark the fully unacceptable, problematic, and acceptable uses of the CLPs.

Fig 1 shows the mean scores in all the conditions. Table 6 shows the mean values and standard errors (SE) in these conditions.

**Table 6:** Syntactic acceptability scores and by-item and by-subject SEs in each condition. In a cell, the first number is the mean, the first number within bracket is the by-item SE, and the second number within bracket is the by-subject SE. Control conditions are marked in grey.

sequence \ CLP	nominal	verbal	temporal
***ans_v+CLP-n***	5.532 (.110 / .098)	6.016 (.064 / .079)	6.011 (.074 / .080)
***ans_CLP***	5.681 (.094 / .101)	4.478 (.201 / .118)	3.984 (.162 / .151)
***ans_CLP-n***	6.573 (.057 / .053)	6.570 (.061 / .073)	6.695 (.043 / .064)
***ans_control***	1.308 (.034 /.052)		
***iso_CLP***	6.486 (.102 / .152)	3.789 (.341 / .223)	2.951 (.209 / .200)
***iso_control***	2.489 (.088 / .210)		

As shown in Fig 1, the syntactic acceptability scores fell into two groups. Some CLPs were uniformly acceptable, having scores around 6 or 7, whereas others were not, having scores below 5. The latter could be further divided into two groups: Around 3 to 5 and below 3. The rectangles in Fig 1 indicate these three groups. Post-questionnaire surveys from 50 randomly chosen participants showed that, a score of 6 or 7 to a sequence indicated confident syntactic acceptability of the sequence to the participants, whereas other scores marked unacceptability of the sequence to the participants; for an unacceptable sequence, a score of 1 or 2 reflected that the participants deemed it as totally unacceptable, and a score of 3 to 5 showed that they viewed it as problematic and were reluctant to accept or use it in practice.

Fig 1 shows that the scores of the nominal CLPs in all the four sequences *ans_v+CLP-n*, *ans_CLP*, *ans_CLP-n*, and *iso_CLP* were around 6 to 7, indicating that the nominal CLPs were uniformly syntactically acceptable to the participants. The scores of *ans_CLP-n* and *iso_CLP* were higher than those of *ans_v+CLP-n* and *ans_CLP*. This could be ascribed to the degrees of simplicity of these sequences: *ans_CLP-n*, as a simplified answer, is more acceptable than *ans_v+CLP-n* and *ans_CLP*, since the latter two types of sequences contain redundant information.

In addition, the scores of *ans_control* and *iso_control* were around 1 or 2, indicating that a sequence without a classifier was by no means acceptable. The score of *iso_control* was slightly higher than that of *ans_control*. This could be ascribed to the contextual effect. As in authoritative Mandarin Chinese grammar books (cf. [12,41–44]), in a situation of informal context or no context, a numeral like *yi* ‘one’ may be omitted as in *Wo mai le (yi) ben shu* ‘I bought (one) CL book’, and a classifier can also be excluded in Mandarin idioms (e.g., *yi ma dang xian* ‘lit. a horse in a leading position’). This might prompt some participants to score *iso_control* slightly higher. Nonetheless, the generally low scores of *iso_control* indicated that independent use of such sequences was largely unacceptable.

Furthermore, the verbal and temporal CLPs in *ans_v+CLP-n* and *ans_CLP-n* were highly acceptable. *ans_CLP-n* are syntactically acceptable by themselves. The presence of a verb in *ans_v+CLP-n* links the verbal or temporal CLP, thus rendering acceptance of such sequences to native speakers as well. The higher scores of *ans_CLP-n* than those of *ans_v+CLP-n* could be due to the higher degree of simplicity of *ans_CLP-n* than that of *ans_v+CLP-n*.

Finally, the scores of the verbal and temporal CLPs in *ans_CLP* and *iso_CLP* were noticeably below 5, indicating that using such sequences is syntactically unacceptable and problematic. The higher scores of *ans_CLP* than those of *iso_CLP* could be due to the sentential context provided by the questions; once the context was removed, the scores dropped significantly, especially the temporal CLPs.

The scores of the verbal and temporal CLPs in *iso_CLP* were different from those of the controls. This could be ascribed to the existence of colloquial expressions. For example, a few verbal or temporal CLPs, though compositionally non-canonical, have been widely used in Mandarin Chinese, e.g., *yi dun fan* ‘lit. one CL: time meal’ or *yi zhen yu* ‘lit. a CL: moment rain’. Albeit such non-canonical yet widely used expressions were not included in the questionnaires, they might stimulate some participants to give slightly higher scores to similar items in the questionnaires. However, the syntactic acceptability scores of independent use of the verbal or temporal CLPs were on average much lower than those of independent use of the nominal CLPs. This indicates that the [temporal or verbal classifier + N] configuration in the above-mentioned expressions (e.g., *yi dun fan* ‘lit. one CL: time meal’ or *yi zhen yu* ‘lit. a CL: moment rain’) has not been schematized into a productive construction as the nominal CLPs.

These results confirmed that the nominal CLPs could be used either independently (*ans_CLP*, *iso_CLP*), in combination with verbs (*ans_v+CLP-n*), or in a simplified way by excluding the nominals (*ans_CLP-n*). This is in agreement with the descriptive grammar of Mandarin Chinese. By contrast, the verbal and temporal CLPs were syntactically unacceptable, no matter whether they were used as answers to questions (*ans_CLP*) or not (*iso_CLP*). They were acceptable only when the verbs were preserved (*ans_v+CLP-n*) or used without the nominals (*ans_CLP-n*). This finding indicates that syntactically the verbal and temporal CLPs are not canonical constituents as the nominal CLPs.

### Semantic acceptability test

After the Greenhouse-Geisser correction, the ANOVA revealed a significant main effect of the CLP type (by-item: *F*(3, 36) = 22.580; *p* < .0001; *η*_*p*_^*2*^ = .653; by-subject: *F*(3, 104) = 49.930; *p* < .0001; *η*_*p*_^*2*^ = .590).

After the Bonferroni correction, the pairwise T-tests showed that except the nominal CLPs, the scores of the verbal and temporal CLPs were not significantly different from those of the controls (see S5 Table). The post-hoc pairwise T-tests showed that the score of the nominal CLPs was significantly higher than those of the verbal or temporal CLPs, and the scores of the verbal and temporal CLPs were not significantly different from each other (see S6 Table).

**Fig 2:**
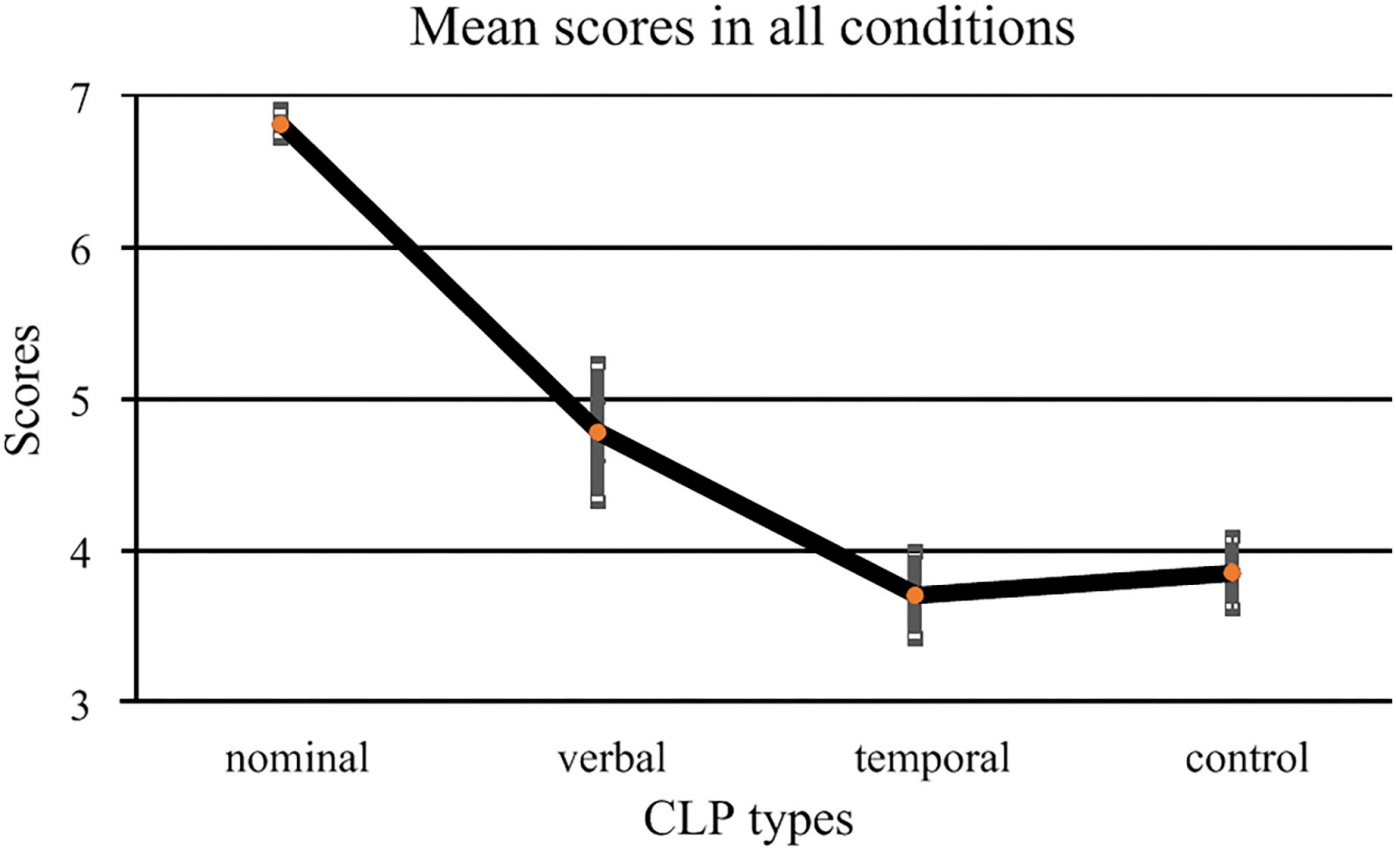
Semantic acceptability scores in each condition. The two error bars at each data point mark the SEs in the by-item and by-subject analyses, respectively.

Fig 2 presents the mean scores in all the four conditions. Table 7 shows the mean values and their SEs in these conditions.

**Table 7:** Semantic acceptability scores and by-item and by-subject SEs in each condition. In a cell, the first number is the mean, the first number within bracket is the by-item SE, and the second number within bracket is the by-subject SE. Control conditions are marked in grey.

sequence \ CLP	nominal	verbal	temporal
***iso_CLP***	6.811 (.098 / .039)	4.785 (.458 / .193)	3.711 (.292 / .245)
***iso_control***	3.859 (.238 / .253)		

Fig 2 shows that the semantic acceptability scores fell into two groups: a score around 6 or 7, indicating uniform acceptability of a sequence; and a score below 5, indicating unacceptability of a sequence.

The scores of the controls were around 4, rather than 1, indicating that the participants could deduce the meanings from the controls, even though the syntactic structures of the controls were obviously unacceptable. This suggests that the participants made use of a criterion different than their syntactic knowledge to judge the acceptability of these expressions. In addition, in terms of semantic acceptability, the verbal and temporal CLPs were not statistically different from each other or the controls. In other words, semantically speaking, the acceptability of all these sequences was similar. Therefore, the (un)grammaticality of those CLPs was not due to semantic factors. Rather, it was the syntactic acceptability scores of those CLPs, as shown in Fig 1, that helped clarify whether those CLPs were proper constituents, compared with the nominal CLPs.

### Constituency test

In order to draw more inferences about the grammatical status of the verbal and temporal CLPs, we also combined our experiment with the constituency test below.

Generative linguists (e.g., [17,45,46]) tend to analyze the verbal and temporal CLPs as valid phrasal constituents and treat them as noun or verb phrases. The examples in Table 8 have been constructed as supportive evidence:

**Table 8:** Examples for constituency test.

(1)	a. *Ta mei shang guo* ***yi tian daxue***. ([17]: 14)
	3SG not go ASP one CL: day college
	‘He has never gone to any college.’
	b. *Ta* ***yi tian daxue*** *mei shang guo*.
	3SG one CL: day college not go ASP
	‘He has never gone to any college.’
(2)	*Ta qu le* ***liang ci Niuyue***, ***san ci Taibei***. ([17]: 21)
	3SG go ASP two CL: time New York three CL: time Taibei
	‘He has been to New York two times, and Taibei three times.’

The non-canonical sequences can move together to a pre-verbal position as shown in example (1b) in Table 8, where the sequence *yi tian daxue* ‘lit. one CL: day college’ appears before the verb *shang* ‘go’. Such sequences can also be conjoined as illustrated by example (2) in Table 8, where the sequences *liang ci Niuyue* ‘lit. two CL: time New York’ and *san ci Taibei* ‘three CL: time Taibei’ are conjoined in the same way as the two noun phrases. Based on such constituency tests, Huang ([17]: 18) claims that such sequences, though combined in a non-canonical fashion, should be treated as *noun phrases* when appearing in *a pre-verbal position* as in example (1b) in Table 8, or *verb phrases* when appearing *in a post-verbal position* as in example (2) in Table 8.

The weakness of Huang’s treatment of the non-canonical CLPs is obvious: How can linguistic expressions with the same configuration as such change their syntactic category if placed in different positions (i.e., sometimes noun phrases and sometimes verb phrases)? Although the whole sentences in Table 8 are fully acceptable, the temporal CLPs such as *yi tian daxue* ‘lit. one CL: day college’ and the verbal CLPs such as *liang ci Niuyue* ‘lit. two CL: time New York’ are not genuine phrases. Rather, they are in the configuration of [pseudo-modifier + noun], that is, the temporal or verbal classifiers syntactically seem to modify the nominal objects (*daxue* ‘college’ in example (1) and *Niuyue* ‘New York’ in example (2) in Table 8), yet semantically quantify the events expressed by the verb phrases (*shang daxue* ‘go to college’ and *qu Niuyue* ‘go to New York’). Here, a question arises: How can we account for the fact that the verbal and temporal CLPs can appear in a pre-verbal position as well as be conjoined? Based on our experiment, we suggest that it should be the sentential context that creates the illusion that such sequences work as proper phrases. Below, we adapted example (2) in Table 8 into four examples (a-d) in Table 9, respectively.

**Table 9:** Four examples (a-d) adapted from example (2) in Table 8. Unacceptable examples are marked by “*”, whose meanings are not shown.

a. *Ta qu le* ***Niuyue liang ci*, *Taibei san ci***.
3SG go ASP New York two time Taipei three time
‘He has been to New York two times, and Taipei three times.’
b. *Ta* ***Niuyue*** *qu le* ***liang ci***, ***Taibei san ci***.
3SG New York go ASP two time Taipei three time
Lit.‘As for New York, he has been to two times, and as for Taipei, three times.’
c. *Ta ***Niuyue liang ci*** *qu le*, *Taibei san ci*.
3SG New York two time go ASP Taipei three time
d. *Ta ***liang ci Niuyue*** *qu le*, *Taibei san ci*.
3SG two time New York go ASP Taipei three time

The numeral and the verbal classifiers *liang ci* ‘two times’, *san ci* ‘three times’ and the nominals *Niuyue* ‘New York’ and *Taibei* in example (2) in Table 8 can swap positions, and the resulting sentence example (a) in Table 9, where *Niuyue liang ci* ‘lit. New York two times’ and *Taibei san ci* ‘lit. Taipei three times’ are also conjoined, is also perfectly felicitous. Note that if the nominal *Niuyue* ‘New York’ moves to a pre-verbal position, it must be separated from the verbal classifier *liang ci* ‘two times’ as in example (b) in Table 9, indicating the loose relation or disassociation between the nominal and the verbal classifier. More importantly, as shown in examples (c) and (d) in Table 9, the resulting sentences would be *ungrammatical* when, the non-canonical sequences, whether in the [nominal + temporal classifier] or [temporal classifier + nominal] configuration, move to a pre-verbal position. This unequivocally constitutes evidence against the status of non-canonical classifier phrases as a phrasal constituent.

## General discussion and conclusion

In this paper, we conducted a psycholinguistic experiment and a theoretical constituency test to analyze whether a particular type of CLPs can stand alone as a canonical phrase. Both the experimental evidence and the constituency test results suggest that the verbal or temporal classifier and the nominal in the verbal or temporal CLPs are not integrated into a proper structural relation, and hence the [temporal/verbal classifier + N] sequence does not form a phrasal constituent, be it noun phrase or verb phrase. In addition, it is the sentential context that makes such non-canonical sequences look like structurally well-formed ones. This is in line with the perspective of some traditional linguists (e.g., [15]), who reasonably treat these pre-nominal temporal or verbal classifiers are *wei dingyu* ‘pseudo-attributives’. Given that the non-canonical classifier sequences demonstrate the gradient nature of grammaticality, we propose that they should be treated as a semi-phrasal category: though structurally more related than arbitrary sequences, the verbal and temporal CLPs are not on the same footing as the nominal CLPs; the nominal and its classifier match each other both semantically and syntactically and hence have a compositionally structural relation, whereas the verbal and temporal CLPs have significantly low syntactic as well as semantic acceptance when standing out of context.

Our experiment reveals potential interactions between those CLPs and other parts of the sentences. With a sentential context, the same temporal or verbal CLPs in distinct expressions (containing a verb or not) (*ans_v+CLP-n* vs. *ans_CLP*) started to show disparate syntactic acceptability scores, compared to the case without the sentential context. In addition, our experiment reveals potential interaction between the syntactic and semantic aspects of those CLPs. For example, Fig 2 shows that the semantic acceptability scores of the nominal CLPs were significantly higher than those of the temporal and verbal CLPs; in other words, a syntactically well-formed sequence also tends to have a higher score in semantic judgment. Both of these aspects suggest that grammatical studies should take a dynamic perspective [2,47] in the sense that we should determine the grammatical status of a sequence of words by examining their interaction with other elements in a sentence and with other linguistic aspects.

Finally, the experimental and statistical approaches adopted in this study and the relevant findings could shed light on previous theoretical linguistic research that relies heavily on intuitive judgments. Intuitive judgments and theoretical discussions from linguists can direct us to particular constructs in different languages, but these intuitive judgments are probably biased due to the knowledge and training background of linguists. Therefore, we need to gather, via experiments, the responses from a general audience without specialized linguistic knowledge towards those constructs. Then, statistical analyses on these responses could complement or even revise intuitive judgments, and comprehensively reflect the natural competence of native speakers towards those constructs. In this sense, theoretical linguists and experimental psychologists should work closely to better understand the grammatical status of a sequence of words.

## Supporting information

S1 Text About Mandarin *de*. (PDF)

S1 Table Links of questionnaires and notes: (a)–(d) for syntactic acceptability, and (e) for semantic acceptability. (PDF)

S2 Table Results of pairwise T-tests of the syntactic acceptability scores between the control and other conditions. In a cell, the first number is by-item *t*-value, and the second by-subject *t*-value. All *p*-values are smaller than .001.
(PDF)

S3 Table Results of pairwise T-tests of the syntactic acceptability scores for comparisons between CLP types. In a cell, the first number is by-item t-value, and the second by-subject *t*-value. Stars indicate *p*-values. Grey cells mark the comparisons having both the by-item and by-subject *p*-values bigger than .001.
(PDF)

S4 Table Results of pairwise T-tests of the syntactic acceptability scores for the comparisons between phrase types. In a cell, the first number is by-item *t*-value, and the second by-subject *t*-value. Stars indicate *p*-values. Grey cells mark the comparison having both the by-item and by-subject *p*-values bigger than .001.
(PDF)

S5 Table Results of pairwise T-tests of the semantic acceptability scores for comparisons between the CLP types and the controls. In a cell, the first number is by-item *t*-value, and the second by-subject *t*-value. Stars indicate *p*-values. Grey cells mark the comparisons having both the by-item and by-subject *p*-values bigger than .001.
(PDF)

S6 Table Results of pairwise T-tests of the semantic acceptability scores for comparisons between the CLP types. In a cell, the first number is by-item *t*-value, and the second by-subject *t*-value. Stars indicate *p*-values. Grey cells mark the comparisons having both the by-item and by-subject *p*-values bigger than .001.
(PDF)
