# Correction: The acceptability judgment of Chinese pseudo-modifiers with and without a sentential context

**DOI:** 10.1371/journal.pone.0221073

**Published:** 2019-08-07

**Authors:** The PLOS ONE Staff

The initials associated with corresponding author Yicheng Wu’s contact information are incorrect. The correct contact information is: wuyicheng@zju.edu.cn (YW). The publisher apologizes for this error.
