# Supplementary material for: The acceptability judgment of Chinese pseudo-modifiers with and without a sentential context
Source: PLoS One. 2019 Jul 18;14(7):e0219896. doi: 10.1371/journal.pone.0219896 (PMC6638940; doi:10.1371/journal.pone.0219896)
Supplement: S1 Text — (PDF) [file pone.0219896.s001.pdf]

S1 Text. About Mandarin *de*

As is well known, the Mandarin *de* is a very problematic morpheme. It is generally considered to have multiple functions depending on different structures in which it occurs. One of its salient functions is that it is often used as an evidential marker, as in example S1(b) below (Cf. S1(a) describes a fact):

(S1) a. *Lanlan shinian qian mai le fang.*

Lanlan ten years ago buy ASP house

'Lanlan bought a house ten years' ago.'

(Note that this sentence is just a statement about when Lanlan bought a house').

b. *Lanlan shinian qian mai de fang.*

Lanlan ten years ago buy DE house

? 'Lanlan did buy a house ten years' ago.'

(The speaker additionally expresses a sense of verification or validation of the statement made by S1(a).

Considering its function of denoting evidentiality that might more or less affect the experiment in our study, we thus excluded it in our materials.

As for its role in determining grammaticality of the relevant structures addressed in our study, comparatively, *de* can occur more naturally in the CLPs containing a temporal expression than those containing a verbal classifier, as in S2(a) and S2(b) below:

(S2) a. 星期六和星期天，我乘车横贯北京城，跑到北京图书馆去看几个小时的书 (王小波《东宫·西宫》)

Xingqiliu he Xingqitian, wo chengche hengguan beijingcheng paodao Beijing Tushucheng,

Saturday and Sunday I take a bus go cross Beijing city arrive Beijing Library
qukan jige xiaoshi de shu.
read several hour DE book
'On Saturdays and Sundays, I took a bus and went across the city of Beijing to Beijing Library, and did some reading for several hours.'

b. 当天 在 书房里 看了 几 小时的书，伯父并没有说什么 (张恨水《北雁南飞》)
Dangtian zai shufangli kanle ji xiaoshi de shu, Bofu bing meiyou shuo shenme.
That day in study read ASP several hour DE book Uncle yet not say what
'That day (I) did some reading in the study room for a few hours, yet my uncle said nothing about it.'

There are two reasons behind this difference. First, a temporal expression (pseudo-modifier) + DE, which seemingly modifies the bare noun yet actually modifies the whole VP (*kanshu* 看书 'read books'), is used to stress the duration of the action or event denoted by the VP. Second, a verbal classifier, by its name, is supposedly used to modify the verb, as *liangci* two 'CL: time' in *changle* (sing) *liangci (*de) ge* (song) and *sanxia* 'three CL: blow' in *dale* (play) *sanxia* (*de) *gu* (drum). Note that, interestingly, some informants reported that the expression with *de* (i.e. *chang le liangci de ge* 'sing ASP two CL: time *de* song') also sounded acceptable to them.

Considering the complexity that *de* might add to the experiment and its optionality (i.e., it is not an obligatory element in the formation of the 'anomalous' structures investigated), we decided not to include it in the current study.
