# Supplementary material for: The acceptability judgment of Chinese pseudo-modifiers with and without a sentential context
Source: PLoS One. 2019 Jul 18;14(7):e0219896. doi: 10.1371/journal.pone.0219896 (PMC6638940; doi:10.1371/journal.pone.0219896)
Supplement: S1 Table — (PDF) [file pone.0219896.s002.pdf]

S1 Table. Links of questionnaires and notes: (a)–(d) for syntactic acceptability, and (e) for semantic acceptability.

Index	Links
(a)	https://docs.google.com/forms/d/1K5CkH4a32aoveXJdd4aAb0_f9Vtt8B9FY0G39w0PjMw/viewform
(b)	https://docs.google.com/forms/d/1fYPLLRXBy0d3wqwPgtHTnZoqI1-i8N09BhHyFWTlqG8/viewform
(c)	https://docs.google.com/forms/d/1dzXg-xrtaCgpj0zFaP_dDxRoZaqohaij0SiZdy3FR00/viewform
(d)	https://docs.google.com/forms/d/1FNv1EdC3ltwHyns0_PRMSFdQtJcKtkX8L43OQQXOvmc/viewform
**Note:** In questionnaires (a)-(d), the English translations of the judgment questions are:<br>"For each of the 10 questions below, please judge the acceptability of the 4 answers after the question according to your knowledge of Mandarin Chinese:<br>1: Syntactically the most irrational or unnatural;<br>…<br>4: Uncertain or neutral;<br>…<br>7: Synoptically the most rational or natural;"	
(e)	https://docs.google.com/forms/d/e/1FAIpQLSfkN93WqeSonTVZnw2kpi46kT8Pwtfr3gaVDgpb7fIiySbOIw/viewform
**Note:** In questionnaire (e), the English translations of the questions are:<br>"For each of the 10 questions below, please judge the acceptability of the 4 answers after the question according to your knowledge of Mandarin Chinese:<br>1: Semantically the most irrational or unnatural;	

…

4: Uncertain or neutral;

…

7: Semantically the most rational or natural;”
