# Supplementary material for: The acceptability judgment of Chinese pseudo-modifiers with and without a sentential context
Source: PLoS One. 2019 Jul 18;14(7):e0219896. doi: 10.1371/journal.pone.0219896 (PMC6638940; doi:10.1371/journal.pone.0219896)
Supplement: S2 Table — In a cell, the first number is by-item t-value, and the second by-subject t-value. All p-values are smaller than .001. (PDF) [file pone.0219896.s003.pdf]

S2 Table. Results of pairwise T-tests of the syntactic acceptability scores between the control and other conditions. In a cell, the first number is by-item *t*-value, and the second by-subject *t*-value. All *p*-values are smaller than .001.

**CLP in sequence \ control**	***ans_control***	***iso_control***
**nominal CLP in *ans_v+CLP-n***	18.954 / 13.865	
**verbal CLP in *ans_v+CLP-n***	30.907 / 16.193	
**temporal CLP in *ans_v+CLP-n***	26.961 / 16.149	
**nominal CLP in *ans_CLP***	25.611 / 13.201	
**verbal CLP in *ans_CLP***	9.216 / 8.526	
**temporal CLP in *ans_CLP***	8.181 / 5.566	
**nominal CLP in *ans_CLP-n***	37.633 / 18.667	
**verbal CLP in *ans_CLP-n***	37.439 / 17.124	
**temporal CLP in *ans_CLP-n***	46.076 / 18.445	
**nominal CLP in *iso_CLP***		40.544 / 31.929
**verbal CLP in *iso_CLP***		7.318 / 9.859
**temporal CLP in *iso_CLP***		7.854 / 7.183
