# Supplementary material for: The acceptability judgment of Chinese pseudo-modifiers with and without a sentential context
Source: PLoS One. 2019 Jul 18;14(7):e0219896. doi: 10.1371/journal.pone.0219896 (PMC6638940; doi:10.1371/journal.pone.0219896)
Supplement: S3 Table — In a cell, the first number is by-item t-value, and the second by-subject t-value. Stars indicate p-values. Grey cells mark the comparisons having both the by-item and by-subject p-values bigger than .001. (PDF) [file pone.0219896.s004.pdf]

S3 Table. Results of pairwise T-tests of the syntactic acceptability scores for comparisons between CLP types. In a cell, the first number is by-item t-value, and the second by-subject *t*-value. Stars indicate *p*-values. Grey cells mark the comparisons having both the by-item and by-subject *p*-values bigger than .001.

**CLP vs. CLP \ sequence**	***ans_v+CLP-n***	***ans_CLP***	***ans_CLP-n***	***iso_CLP***
**nominal vs. verbal**	-4.679*** / -5.660***	6.853*** / 10.343***	0.043 / 0.042	6.955*** / 10.798***
**verbal vs. temporal**	0.057 / 0.070	4.971*** / 4.338***	-2.016$^{\#}$ / -2.132 *	2.297* / 8.339***
**nominal vs. temporal**	-3.093* / -5.987***	9.819*** / 10.792***	-4.538*** / -1.712$^{\#}$	13.073*** / 14.390***

Note: *** $p< .001$; ** $p< .01$; * $p< .05$; # $p< .1$
