# Supplementary material for: The acceptability judgment of Chinese pseudo-modifiers with and without a sentential context
Source: PLoS One. 2019 Jul 18;14(7):e0219896. doi: 10.1371/journal.pone.0219896 (PMC6638940; doi:10.1371/journal.pone.0219896)
Supplement: S4 Table — In a cell, the first number is by-item t-value, and the second by-subject t-value. Stars indicate p-values. Grey cells mark the comparison having both the by-item and by-subject p-values bigger than .001. (PDF) [file pone.0219896.s005.pdf]

S4 Table. Results of pairwise T-tests of the syntactic acceptability scores for the comparisons between phrase types. In a cell, the first number is by-item *t*-value, and the second by-subject *t*-value. Stars indicate *p*-values. Grey cells mark the comparison having both the by-item and by-subject *p*-values bigger than .001.

**phrase vs. phrase \ CLP**	**nominal**	**verbal**	**temporal**
ans_v+CLP-n* vs. *ans_CLP	-0.859 / -1.246	6.450*** / 14.479***	9.840*** / 12.503***
ans_CLP* vs. *ans_CLP-n	-7.901*** / -8.380***	-10.398*** / -15.647***	-15.415*** / -17.192***
ans_v+CLP-n* vs. *ans_CLP-n	-8.313*** / -10.636***	-4.892*** / -5.131***	-6.557*** / -6.639***
ans_CLP* vs. *iso_CLP	-4.987*** / -4.319***	3.162** / 2.849**	4.812*** / 4.324***

Note: *** $p< .001$; ** $p< .01$; * $p< .05$; # $p< .1$.
