# Supplementary material for: The acceptability judgment of Chinese pseudo-modifiers with and without a sentential context
Source: PLoS One. 2019 Jul 18;14(7):e0219896. doi: 10.1371/journal.pone.0219896 (PMC6638940; doi:10.1371/journal.pone.0219896)
Supplement: S5 Table — In a cell, the first number is by-item t-value, and the second by-subject t-value. Stars indicate p-values. Grey cells mark the comparisons having both the by-item and by-subject p-values bigger than .001. (PDF) [file pone.0219896.s006.pdf]

S5 Table. Results of pairwise T-tests of the semantic acceptability scores for comparisons between the CLP types and the controls. In a cell, the first number is by-item *t*-value, and the second by-subject *t*-value. Stars indicate *p*-values. Grey cells mark the comparisons having both the by-item and by-subject *p*-values bigger than .001.

**CLP \ control**	***iso_control***
**nominal**	10.738*** / 10.922***
**verbal**	2.265** / 3.689**
**temporal**	-.443 / -.591

Note: *** $p< .001$; ** $p< .01$; * $p< .05$; # $p< .1$.
