# Supplementary material for: The acceptability judgment of Chinese pseudo-modifiers with and without a sentential context
Source: PLoS One. 2019 Jul 18;14(7):e0219896. doi: 10.1371/journal.pone.0219896 (PMC6638940; doi:10.1371/journal.pone.0219896)
Supplement: S6 Table — In a cell, the first number is by-item t-value, and the second by-subject t-value. Stars indicate p-values. Grey cells mark the comparisons having both the by-item and by-subject p-values bigger than .001. (PDF) [file pone.0219896.s007.pdf]

S6 Table. Results of pairwise T-tests of the semantic acceptability scores for comparisons between the CLP types. In a cell, the first number is by-item *t*-value, and the second by-subject *t*-value. Stars indicate *p*-values. Grey cells mark the comparisons having both the by-item and by-subject *p*-values bigger than .001.

**CLP vs. CLP \ phrase**	***iso_CLP***
**nominal vs. verbal**	5.475*** / 10.922***
**nominal vs. temporal**	9.102*** / 12.581***
**verbal vs. temporal**	2.482* / 3.732**

Note: *** $p< .001$; ** $p< .01$; * $p< .05$; # $p< .1$.
